# A COVID-19 Test Triage Tool, Predicting Negative Results and Reducing the Testing Burden on Healthcare Systems During a Pandemic

**DOI:** 10.3389/fmed.2021.563465

**Published:** 2021-04-29

**Authors:** Dara J. Lundon, Brian D. Kelly, Sujit Nair, Damien M. Bolton, Gopi Patel, David Reich, Ashutosh Tewari

**Affiliations:** ^1^Department of Urology, Icahn School of Medicine, Mount Sinai Hospitals, New York, NY, United States; ^2^Department of Urology, Austin Health, Melbourne, VIC, Australia; ^3^Department of Infectious Diseases, Icahn School of Medicine, Mount Sinai Hospitals, New York, NY, United States; ^4^Department of Anesthesiology, Perioperative and Pain Medicine, Icahn School of Medicine at Mount Sinai, New York, NY, United States

**Keywords:** COVID-19, SARS–CoV-2, risk prediction, clinical decision aid, resource allocation

## Abstract

**Background:** Detecting and isolating cases of COVID-19 are amongst the key elements listed by the WHO to reduce transmission. This approach has been reported to reduce those symptomatic with COVID-19 in the population by over 90%. Testing is part of a strategy that will save lives. Testing everyone maybe ideal, but it is not practical. A risk tool based on patient demographics and clinical parameters has the potential to help identify patients most likely to test negative for SARS-CoV-2. If effective it could be used to aide clinical decision making and reduce the testing burden.

**Methods:** At the time of this analysis, a total of 9,516 patients with symptoms suggestive of Covid-19, were assessed and tested at Mount Sinai Institutions in New York. Patient demographics, clinical parameters and test results were collected. A robust prediction pipeline was used to develop a risk tool to predict the likelihood of a positive test for Covid-19. The risk tool was analyzed in a holdout dataset from the cohort and its discriminative ability, calibration and net benefit assessed.

**Results:** Over 48% of those tested in this cohort, had a positive result. The derived model had an AUC of 0.77, provided reliable risk prediction, and demonstrated a superior net benefit than a strategy of testing everybody. When a risk cut-off of 70% was applied, the model had a negative predictive value of 96%.

**Conclusion:** Such a tool could be used to help aide but not replace clinical decision making and conserve vital resources needed to effectively tackle this pandemic.

## Introduction

Detecting and isolating cases of COVID-19 are amongst the key elements listed by the WHO to reduce transmission (1). While everybody is at risk of infection, the majority of the population has not been tested. It has been reported that blanket testing and isolation of positive cases in a village in Italy with a population of ~3,000 people, saw the number of people with COVID-19 symptoms fall by over 90% in 10 days (2).

While initially there were testing capacity constraints in the USA, Government officials reported that there would be a much greater supply and availability of testing (3). However, now over a month since such assurances, there are still reports of shortages of testing for SARS-CoV-2 (4–6). While a PCR test of a nasopharyngeal swab or serum antibody test are now standards for COVID-19 diagnosis, providing this on a population level is neither practical nor necessary. The decision of whom to test is further complicated by the fact that this pandemic overlaps with seasonal flu; a syndrome which presents with many similar features to COVID-19.

However, a freely available and rapid risk assessment, which provides the likelihood of a positive test, may help provide the assurances many need, and encourage many more to continue to isolate themselves in their individual effort to reduce further SARS-CoV-2 transmission.

Risk calculators have been widely used in clinical practice for many years, particularly in urological cancers to help identify those at risk of a cancer diagnosis and also to correctly identify those at risk of a high risk cancer diagnosis (7, 8). Given the range of symptoms that patients with COVID-19 present with to hospitals in the US, we postulated that a novel risk calculator may aid in the risk assessment and triaging of these patients to help identify those at risk of being diagnosed with COVID-19 to help tailor their medical care. As this pandemic continues, all US hospitals will be under considerable pressure to maintain a high standard of care and a novel risk calculator may aide in the initial triaging of these patients.

## Methods

### Patient Data

De-identified patient data was obtained from the Mount Sinai Healthcare System (MSHS) Data Warehouse (https://msdw.mountsinai.org/) for all patients who present for testing prior to April 4th 2020 (*n* = 21,790). This includes data from 10 institutes and facilities across four boroughs of New York City. Of these, 9,516 having been assessed as meeting state and institutional criteria, underwent testing for SARS-CoV-2 as they were suspected of having COVID-19 (9). There were 4,640 who tested positive following either an oro or naso-pharyneal swab and test with Cobas COVID-19 (Roche Diagnostics, NJ, USA), or CDC COVID-19 assay.

Of this dataset, 4,187 had complete information available on the following variables: age, gender, race, BMI, reported maximum temperature, temperature at time of review, smoking status and comorbidities such as asthma, COPD, hypertension and diabetes.

The MSHS Ethics Committee approved a waiver of documentation of informed consent for use of the; de-identified patient data was obtained from the MSHS Data Warehouse (https://msdw.mountsinai.org/).

### Statistical Analysis

#### Data Analysis

All analyses were performed using R software (10). Continuous data were presented as median [interquartile range (IQR)]. Categorical data were presented as number (percentage). The χ^2^-test was used to compare differences in clinical outcomes between SARS-CoV-2 test Positive and SARS-CoV-2 test negative groups. The dataset was randomly divided with 80% used to develop the prediction model, and 20% maintained as a holdout dataset. Correlation of available demographic and clinical covariates was performed. Using the available demographic and clinical covariates, and a proprietary prediction platform which also captures non-linear interactions, we developed a model to predict a positive SARS-CoV-2 test in the training set, which also used an iterative strategy to select a subset of the available predictors. This produced multiple models which were then validated in the holdout data set. The model which required the least covariates and which was not statistically significant from the model with the greatest discriminative ability in the holdout dataset was chosen.

Classification metrics of the model in the holdout dataset were calculated and include model sensitivity (recall), specificity, accuracy, positive predictive value (precision), and negative predictive value. We assessed the discrimination of the models with the area under the receiver operating characteristic curve [AUC (95% CI)]. AUC values for the various models were compared using U-statistics (11). Calibration curves were computed by comparing observed proportions of a positive SARS-CoV-2 test to mean calculated risks from the model in the holdout cohort. Decision curve analysis was performed to assess for the gain derived from using this model in the holdout cohort over the corresponding net benefit curves of testing all of these patients, or none of these patients (12).

Performance metrics of the model in the hold out set were calculated and tabularized, based on a cut points of 0.7, derived from the training dataset; the point at which negative predictive value was optimal.

## Results

Of the 4,187 patients with complete data, 2,022 (48%) had tested positive for SARS-CoV-2 and 2,165 (52%) had tested negative. The training dataset had 3,349 patients with complete data; of which 1,612 (48%) had a positive SARS-CoV-2 test. Of 838 patients in the holdout dataset, 410 (49%) had a positive SARS-CoV-2 test, and 428 (51%) had a negative SARS-CoV-2 test (Table 1).

**Table 1:** Characteristics of all patients; subdivided by the result of Covid-19 testing.

	**COVID-19 test**								
**Result**	**Positive**			**Negative**			**Total**		
***N***	2022			2165			4187		
	**Quartile**			**Quartile**			**Quartile**		
	**Mean**	**1st**	**3rd**	**Mean**	**1st**	**3rd**	**Mean**	**1^**st**^**	**3rd**
Age (Mean; IQR)	60.2	48.3	73.0	53.0	35.0	68.0	56.6	41.0	71.0
BMI	28.5	24.4	32.0	27.0	22.6	30.6	27.7	23.5	31.3
Temperature on presentation	99.3	98.2	100.3	98.4	97.8	98.8	98.8	97.9	99.5
Maximum temperature	100.7	99.2	102.1	99.4	98.3	100.1	100.0	98.6	101.4
	***N***	%		***N***	%		***N***	%	
Gender (% Male)	1,086	53.7%		893.0	41.2%		1,979	47.3%	
White	539	26.7%		758.0	35.0%		1,297	31.0%	
Black	569	28.1%		577.0	26.7%		1,146	27.4%	
Asian	87	4.3%		117.0	5.4%		204	4.9%	
Other	827	40.9%		713.0	32.9%		1,540	36.8%	
Current smoker	91	4.5%		311.0	14.4%		402	9.6%	
Asthma	132	6.5%		228.0	10.5%		360	8.6%	
COPD	76	3.8%		115.0	5.3%		191	4.6%	
HTN	760	37.6%		609.0	28.1%		1,369	32.7%	
Diabetes	544	26.9%		413.0	19.1%		957	22.9%	
HIV positive	62	3.1%		82.0	3.8%		144	3.4%	
Cancer diagnosis	191	9.4%		325	15.0%		516	12.3%	

**Figure 1:**
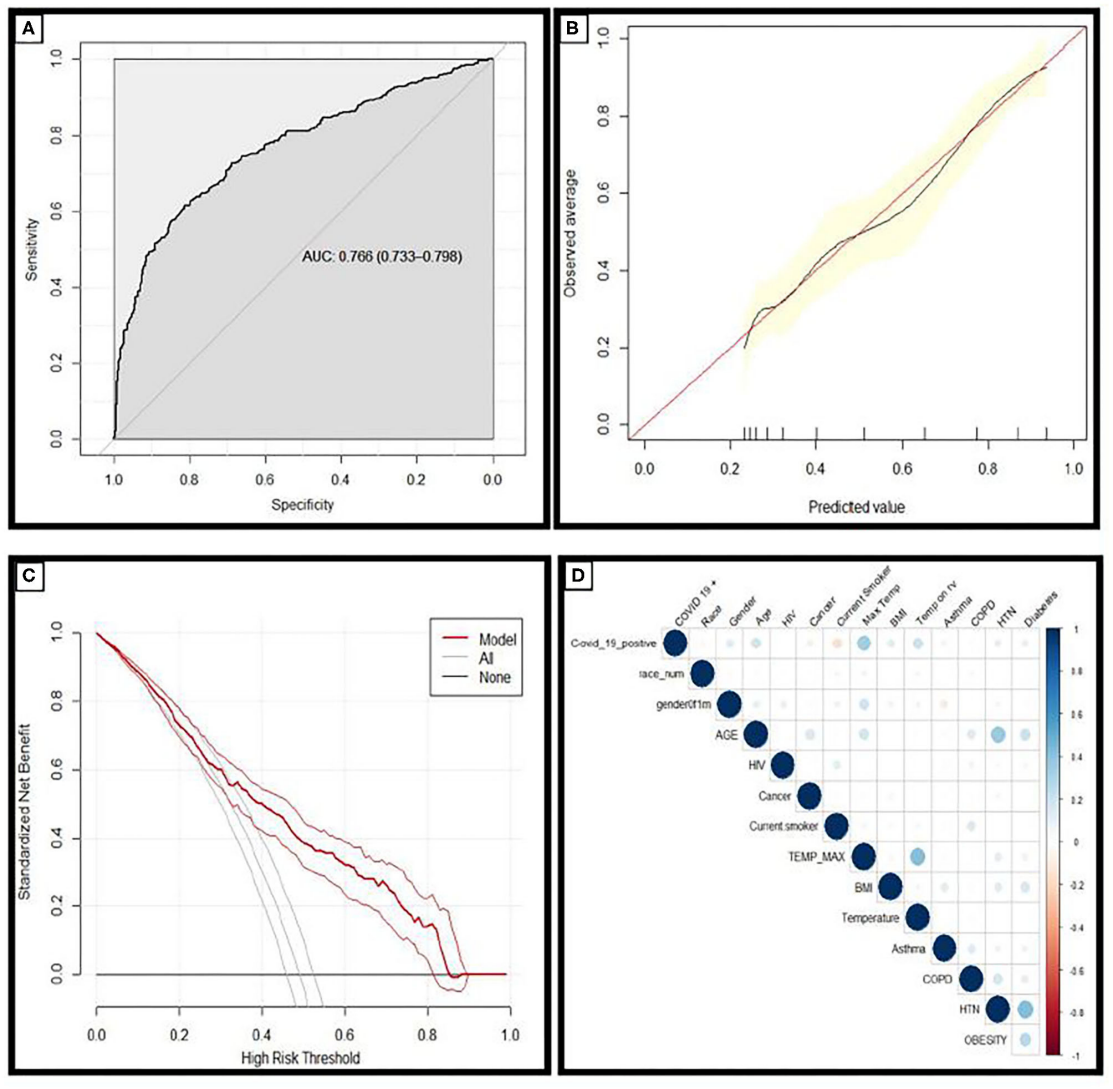
**(A)** Receiver operating characteristic curves and AUC values for the model in the holdout dataset demonstrating the discriminative ability of this model (AUC = 0.766; 95% CI 0.733–0.798). **(B)** Calibration curves of the model in the holdout dataset demonstrating the agreement between predicted and observed probabilities of a positive Covid-19 test. **(C)** Decision curve analysis for positive Covid-19 test prediction in the holdout dataset. **(D)** Correlation matrix demonstrating the correlation between risk factors and COVID-19 diagnosis: the larger the size, and the stronger the color, the higher the correlation.

The AUC value for prediction of a positive SARS-CoV-2 test was 0.77 for this model in the holdout dataset (Figure 1A). A cut-off point of calculated risk was chosen so as to optimize the negative predictive rate and the reduction in SARS-CoV-2 tests. At this cut-off point, the negative predictive value is 96%, and positive predictive value is 66%. Using a risk of 70% in the hold-out dataset would have reduced testing volume by 76%; or 636 tests out of 838, and 91 patients with a positive SARS-CoV-2 test would have been misclassified as negative by this model (Table 2).

**Table 2:** Model performance characteristics for the holdout dataset.

	***N***		**%**	
Presented for testing	838		100%	
Prevalence of COVID-19	410		49%	
	**Risk threshold applied**			
	**0%**		**70%**	
	***N***	**%**	***N***	**%**
Tests performed (n)	838	100%	202	24%
False negatives (n)	0	–	215	26%
False positives (n)	0	–	7	0.8%
Tests avoided *n* (%)	0	–	636	76%
Sensitivity (%)		100%		99%
Specificity (%)		–		48%
PPV (%)		49%		53%
NPV (%)		^†^		92%

*A risk threshold of 0% represents current practice; where everybody in this cohort underwent testing. The optimal cut point for this risk tool which optimized the negative predictive value (NPV) and tests avoided; was 70%. The combination of these criteria maximally reduce the number of total tests, while minimizing the number of COVID-19 positive patients, who are predicted as negative*.

† *Indeterminate t*.

*PPV, Positive Predictive Value; NPV, Negative Predictive Value*.

Calibration plots provide a visual representation of how reliable the predicted risk estimate is; the accuracy of risk estimates relating to the agreement between estimated and observed events.^4^ A curve close to the diagonal indicates that predicted risks correspond well to observed proportions; Figure 1B demonstrates the model has excellent overall calibration. Some overestimation of the risk of a positive test for those with a positive SARS-CoV-2 test is seen for those with calculated risks ~<50% and some underestimation of risk for those with higher calculated risks.

Decision curve analysis was performed and is plotted in Figure 1C. The straight black line at y = 0 represents the net benefit derived from employing a strategy of testing nobody and the gray line represents the net benefit if a strategy of testing everybody was employed. The model was superior to both of these strategies across the entire range of clinically useful threshold risks. The relationship between COVID-19 diagnosis and each risk factor included in this analysis is described in the correlation matrix Figure 1D.

**Figure 2:**
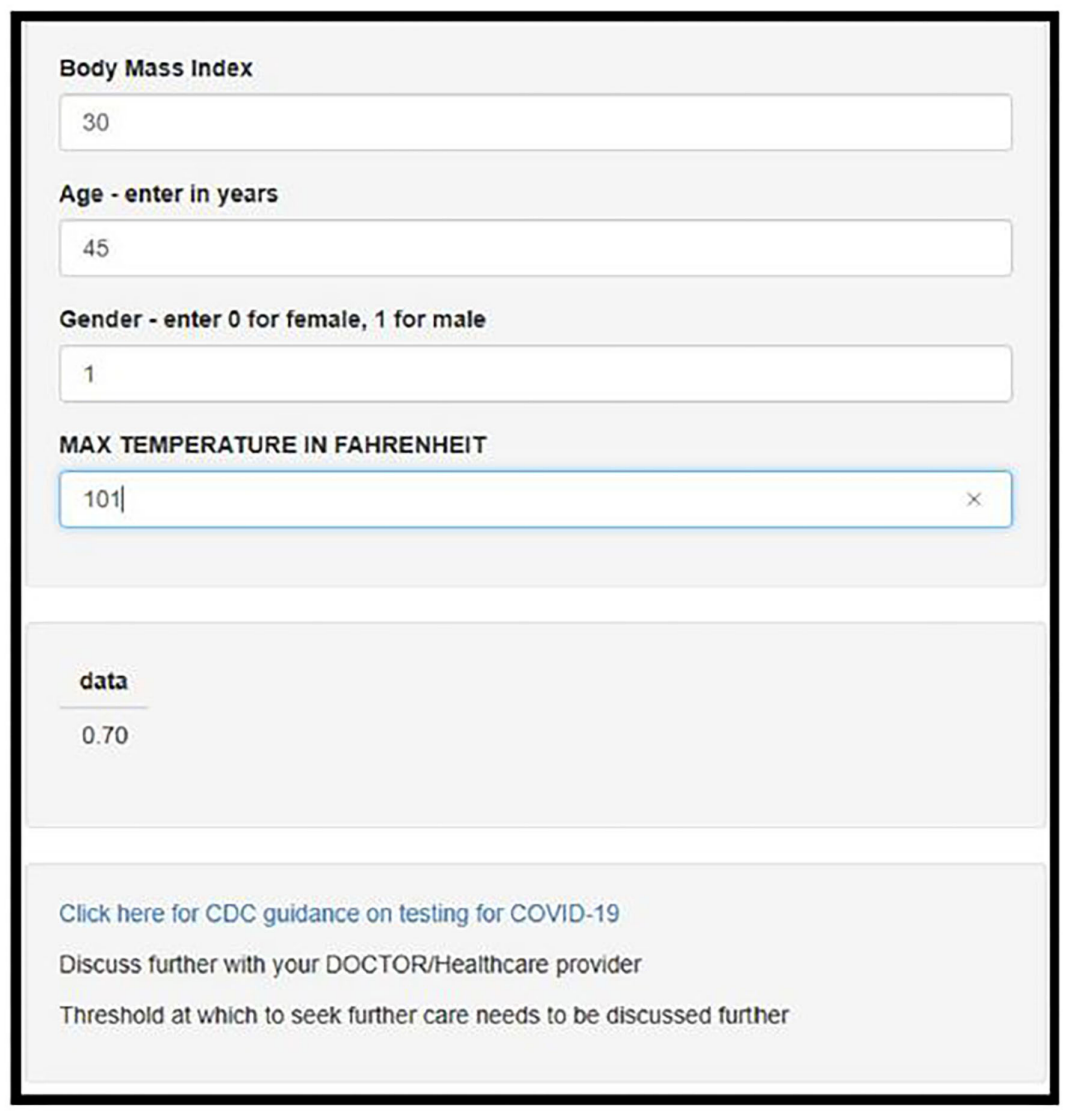
Screenshot of the deployed web-app which is freely available for use online at https://darasriskcalculators.shinyapps.io/Covid_app_x4/.

The model was deployed using online using Shiny, an interactive web based application framework for R statistical software (see Figure 2) (13).

## Discussion

To our knowledge, we are the first in the world to develop and describe such a risk tool to predict the probability of a positive test for SARS-CoV-2. As this pandemic continues to progress and our healthcare system is put under increasing pressure, this novel, world-first risk tool has the potential to aide clinicians, patients and healthcare systems in the response to the COVID-19 pandemic.

### The Value of This Risk Tool and the Significance of Negative Predictive Value Being ~96%

The value of this model, is that not every patient that presents with symptoms suggestive of COVID-19 will test positive; neither will every patient that tests positive require inpatient hospital care (14). While up to 18% of patients infected with SARS-CoV-2 remain asymptomatic, most who do become symptomatic and test positive, will be discharged to their home (15), and only certain patients will need admission (16).

This risk tool could be used as an aide for triage, initial assessment or the first telehealth visit; patients who receive a result of >0.7 (70%) are most likely to test positive for SARS-CoV-2. If they are otherwise well, they can continue to be managed at home, and observe protocols to reduce transmission to others. Follow up telehealth visits can be arranged as necessary.

If a patient receives a result of <0.7 (<70%), they are most likely to test negative for SARS-CoV-2; our results demonstrate that 96% patients in the holdout dataset did in fact test negative (see NPV in Table 2). They can be reassured that they do not need to increase their risk for getting infected by leaving their home unnecessarily. As patients in this cohort did present with minimal criteria for a suspected diagnosis, it is unlikely that their symptoms are due to a COVID-19 diagnosis. Their symptoms therefore are more likely due to another cause such as an underlying ailment, seasonal allergies or influenza. Amidst this pandemic, it is important to remember that influenza and other infections and conditions are still claiming lives. Influenza has a mortality rate of 2/100,000 (17), and the management of patients who are otherwise not in respiratory distress and hemodynamically stable, is, similar to COVID-19: symptomatic management at the patient's home, liaising with their primary care physician and healthcare team.

The negative predictive value of this model is 96% when a threshold risk of 70% is applied. This compares favorably with other widely used medical diagnostics such as standardized extended pattern template biopsy of the prostate, which has a NPV of 25–31% (18), and ultrasound and CT for suspected appendicitis which have respective NPVs of 76 and 95% (19).

### The Significance of the Positive Predictive Value Being 66%

At a threshold risk of 70%, this risk tool has a positive predictive value of 66%: meaning that for every 100 people told that they are likely to have a positive test, 34 will not test positive for Covid-19, while 66 will test positive. The management for many in this group, will be at home; where they should reduce social contacts, and continue transmission mitigation efforts while closely monitoring symptoms and signs. The 47 who will not test positive, will benefit from these measures and help to further flatten the curve. The PPV of this risk tool is greater than the PPV of the criteria applied; the clinical suspicion which led to the COVID-19 test was 49% in the holdout cohort (see 0% risk threshold in Table 2). Placing this figure into context, the PPV of a PSA of 4 ng/ml to predict prostate cancer is ~14–37%, and mammography has a PPV of between 3.3 and 28.6% (20, 21).

The net impact of this is best demonstrated on the decision curve analysis (Figure 1C): Where you can see the net benefit of using this score is greater than the strategies of testing everybody, and testing nobody. It is an informed decision aide, and offers an alternative to the strategy of testing everybody in person, or the strategy of testing nobody at all (Figure 1C).

### Reduce the Burden on Healthcare System, and Reduce Unnecessary Exposures to Patients and Healthcare Workers

As the extent of SARS-CoV-2 continues to spread, other regions and healthcare facilities will come under increased strain on available resources including assessing patients to ensure they warrant testing, administering, transporting, performing and reporting these tests; all of which place patients and healthcare workers at an increased risk of infecting others or being infected. The effectiveness of PPE and prophylaxis was estimated to be ~79% during a previous outbreak (22). We have demonstrated that at a risk cut-off of 70%, testing volume can be reduced by 75%. Using such as solution as this as a triage aide, cannot just reduce the testing burden but in doing so, reduce the exposure risk to patients by reducing unnecessary visits to testing sites.

### Algorithms Are Becoming More Used in Clinical Practice

The use of such a risk tool would not be unprecedented. Numerous algorithms have recently received regulatory approval for broad clinical use (23). The WAVE Clinical Platform is an early warning system integrating real-time vital sign data to identify hospital inpatients who are at risk of vital sign instability and was approved by the FDA in 2018. Since then the FDA has granted clearance for similar algorithms in a number of fields including diagnostics. The successful implementation of such tools has also been documented in other healthcare systems such as the National Health Service in the UK (24). Risk tools to identify those most likely to require ventilatory support or intensive care unit admission would also be of support to clinical decision making.

### Does Patient Behavior Change When They Know They Test Positive for an Infectious Disease?

Behavior changes associated with a positive test for other infectious diseases has been well-documented (25–28). Risk perception of infection has been demonstrated to be a predictor of a range of preventative behaviors. SARS-CoV-2 is unlike influenza in that there are no mechanisms currently available for SARS-CoV-2 prophylaxis. However, in a study on perceptions related to Avian Influenza, Lau et al. identified that those who perceived they had symptoms similar to influenza were more than four times as likely to wear a face mask, than those who did not (29).

Rudisill et al. documented behavioral changes during the course of the H5N1 avian influenza pandemic; noting that residence in a nation in which H5N1 had been found in humans had a significant and positive relationship with being less likely to consume poultry, eggs and egg-related products, whereas in nations where H5N1 was present, but not in humans, there was limited influence on these behaviors (30).

In the absence of PCR and antibody testing for SARS-CoV-2, an approach such as a freely available online risk tool, could be used to help reinforce compliance with positive behaviors which are associated with reduced transmission of SARS-CoV-2.

### Limitations

This dataset is comprised of a cohort of patients with an encounter at a single healthcare system's facility in NYC, who were screened for SARS-CoV-2 infection, and only cases where complete data was available were included (44% of cohort). Therefore, there is an inherent selection bias in this dataset; and in effect it should be used in a similar cohort: those cohorts with a similar social and economic demographic composition to the greater New York City catchment area, who are symptomatic or high risk for COVID-19 infection and presenting to a US healthcare institution. The molecular based assays used in this cohort to diagnose SARS-CoV-2 infection while used widely, are not used universally; and have sensitivity and specificities of >95%; it should be noted that rapid antigen based tests are also used in this setting, and while highly specific, on average they have a lower sensitivity (56%) (31). It is important to note that such a risk tool is for use in symptomatic patients, and does not address those patients who would test positive for COVID-19 but remain asymptomatic. It has been designed as an aide to clinical decision making, and not a replacement for it.

As the pandemic continues to overwhelm healthcare systems, risk tools such as the one described in this paper have the potential to aide clinicians and healthcare institutions. They can more accurately risk stratify patients, identifying those most likely to test positive, and those most likely to test negative for SARS-CoV-2 and aide in the strategic allocation of testing resources and the response of health systems to this pandemic.

## Conclusion

We have developed a risk model which can predict the outcome of SARS-CoV-2 testing which is accurate and reliable. It offers an instant quantification of risk, and is available online for free. Such a tool could be used to help improve compliance with transmission mitigation strategies, reduce the workload and burden on health systems and help reduce exposures associated with unnecessary visits to testing sites.

## Data Availability Statement

The data analyzed in this study is subject to the following licenses/restrictions: De-identified dataset was made available by the Mount Sinai Scientific Computing. Requests to access these datasets should be directed to Ashutosh Tewari, ash.tewari@mountsinai.org.

## Ethics Statement

The studies involving human participants were reviewed and approved by Mount Sinai Hospital Ethics Committee. Written informed consent for participation was not required for this study in accordance with the national legislation and the institutional requirements.

## Author Contributions

DL and AT: concept, literature search, study design, data analysis, data interpretation, writing, and figures. BK: concept, literature search, study design, data analysis, data interpretation, and writing. SN: literature search and data interpretation. DB: data interpretation and writing. GP and DR: literature search, data interpretation, and writing. All authors contributed to the article and approved the submitted version.

## Conflict of Interest

DR is President and Chief Operating Officer of The Mount Sinai Hospital and President of Mount Sinai Queens. The remaining authors declare that the research was conducted in the absence of any commercial or financial relationships that could be construed as a potential conflict of interest.
